# Electroenzymatic Nitrogen Fixation Using a MoFe Protein System Immobilized in an Organic Redox Polymer

**DOI:** 10.1002/anie.202007198

**Published:** 2020-07-22

**Authors:** Yoo Seok Lee, Adrian Ruff, Rong Cai, Koun Lim, Wolfgang Schuhmann, Shelley D. Minteer

**Affiliations:** ^1^ Department of Chemistry University of Utah 315 S 1400 E Salt Lake City Utah 84112 USA; ^2^ Analytical Chemistry—Center for Electrochemical Sciences (CES) Faculty of Chemistry and Biochemistry Ruhr University Bochum Universitätsstr. 150 44780 Bochum Germany; ^3^ Department of Chemistry University of California Berkeley California 94720 USA

**Keywords:** ammonia, bioelectrosynthesis, neutral red, nitrogenase, redox polymers

## Abstract

We report an organic redox‐polymer‐based electroenzymatic nitrogen fixation system using a metal‐free redox polymer, namely neutral‐red‐modified poly(glycidyl methacrylate‐*co*‐methylmethacrylate‐*co‐*poly(ethyleneglycol)methacrylate) with a low redox potential of −0.58 V vs. SCE. The stable and efficient electric wiring of nitrogenase within the redox polymer matrix enables mediated bioelectrocatalysis of N_3_^−^, NO_2_^−^ and N_2_ to NH_3_ catalyzed by the MoFe protein via the polymer‐bound redox moieties distributed in the polymer matrix in the absence of the Fe protein. Bulk bioelectrosynthetic experiments produced 209±30 nmol NH_3_ nmol MoFe^−1^ h^−1^ from N_2_ reduction. ^15^N_2_ labeling experiments and NMR analysis were performed to confirm biosynthetic N_2_ reduction to NH_3_.

## Graphical abstract

**Fixed in the matrix**: An electroenzymatic ammonia‐producing system is demonstrated using nitrogenase immobilized within a specifically designed neutral‐red‐modified redox polymer. Efficient electric wiring of nitrogenase in the redox polymer matrix enabled mediated bioelectrocatalysis and thereby facilitated biosynthetic N_2_ fixation in the absence of ATP and Fe protein.

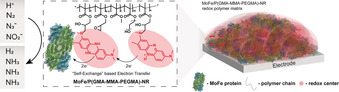

## Introduction

**Figure 1:**
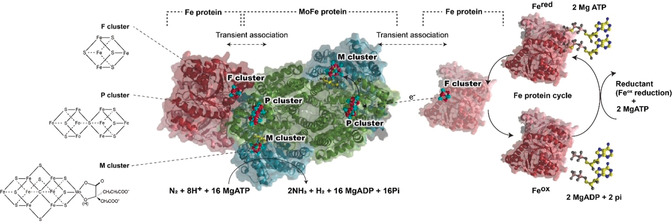
Crystal structure of Mo‐dependent nitrogenase (PDB: 4WZA) from *Azotobacter vinelandii* consists of Fe protein embedding the F cluster and MoFe protein embedding the P cluster and M cluster, illustrating N_2_ turnover in vivo with the expense of repeated steps referred to as the Fe protein cycle. The Fe protein cycle involves 1) a transient association of Fe protein with MoFe protein to concurrently hydrolyze 16 MgATP, and 2) a reduction of the oxidized Fe protein by a reductant, and 3) a replacement of 16 MgATP.

Electronic wiring of biocatalysts to electrodes is critical in advancing current innovations in bioelectrocatalysis including biosensors, biofuel cells, and bioelectrosynthesis systems.^1^, ^2^, ^3^ Nitrogenase gained attention because it catalyzes the reduction of atmospheric N_2_ to NH_3_, breaking the N−N triple bond at physiological pH, room temperature, and ambient pressure.^4^ Specifically, molybdenum‐dependent nitrogenase contains two highly O_2_‐sensitive subunits: a reducing protein (Fe protein) involving the F cluster and a catalytic protein (MoFe protein) including the P cluster and the FeMo‐cofactor.^5^ Biosynthesis of NH_3_ in vivo is energetically expensive with a cost of 16 ATP molecules per reduced N_2_ molecule [Eq. (1)] with transient binding of the Fe protein (≈66 kDa homodimer) to reduce the MoFe protein (≈240 kDa dimer of dimers) (Figure 1 and Figure S1).^6^, ^7^, ^8^, ^9^

(1) $$N{}_{2}+8\;H{}^{+}+8\;e{}^{-}+16\;\mathrm{MgATP}\to 2\;\mathrm{NH}{}_{3}+H{}_{2}+16\;\mathrm{MgADP}+16\;P{}_{i}$$

Thus, there is considerable interest in utilizing electrons from an electrode as the reducing reagent to achieve sustainable NH_3_ formation by nitrogenase without the need for ATP. Several strategies for using electrons from the electrode as a reductant are investigated, each with unique advantages.

An innovative approach for electrodes as electron donors is to directly communicate with the MoFe protein through the tunneling of electrons between the electrode and the enzyme cofactor.^10^ For this, pyrene‐functionalized linear poly(ethyleneimine) was used to immobilize the MoFe protein at a carbon electrode surface to enable direct bioelectrocatalysis, allowing direct bioelectrosynthetic reduction of N_2_ to NH_3_ in the absence of ATP. This enabled the investigation of the direct electrochemical kinetics for each of the cofactors in nitrogenase under biologically relevant conditions.^11^ Electron tunneling theory suggests that direct electron transfer requires the correct orientation between the electron donor and the acceptor within 14 Å distance for efficient electron tunneling,^12^, ^13^ enabling electron transfer to be faster than the enzymatic reaction, thus promoting superior performance.^11^, ^14^ Accordingly, the measured bioelectrocatalytic current is solely due to the enzyme attached as a protein monolayer within the electron tunneling distance.^2^, ^10^, ^14^, ^15^, ^16^

An approach to improve the loading of wired nitrogenase on electrodes is to form an electron‐conducting matrix to entrap and stabilize nitrogenase at the electrode interface by employing redox relays tethered to a polymer network, which are in principle able to efficiently wire any orientation of a redox enzyme to the electrode. Hence, electron transfer (ET) becomes independent of the electrode–enzyme distance and orientation via a mediated charge transfer.^17^, ^18^, ^19^, ^20^, ^21^ The potential of a redox polymer to electrically communicate with nitrogenase via the MoFe protein should be <−0.49 V vs. SCE.^4^, ^5^, ^11^ Accordingly, there are only a few potential redox mediators that could provide a sufficient reducing power, such as a variety of viologen‐modified polymers,^22^, ^23^, ^24^ which offer sufficient reducing power with redox potentials of around −0.69 V vs. SCE. However, the low stability and the consequential unsustainable electron‐conducting relay of the polymer matrix handicapped the further use of the viologen‐modified redox polymers.^25^ Moreover, redox polymers capable of mediated bioelectrocatalysis with nitrogenase have been rarely described due to the complexity of the cofactors in the enzyme.^26^, ^27^

**Figure 2:**
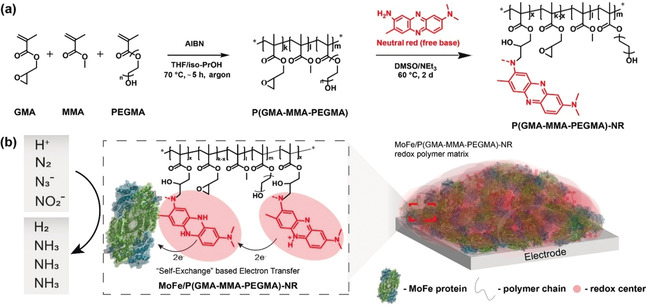
a) Multistep synthesis of the redox polymer P(GMA‐MMA‐PEGMA)‐NR. b) Immobilized nitrogenase bioelectrocatalysis using a redox polymer as an immobilization matrix and an electron mediator.

Here, we report the development of a neutral red (NR) modified poly(glycidyl methacrylate‐*co*‐methylmethacrylate‐*co*‐poly(ethyleneglycol)methacrylate) P(GMA‐MMA‐PEGMA)‐NR based nitrogenase system to enable the stabilization and efficient electric wiring of nitrogenase within an all‐organic redox‐active polymer on the electrode surface (Figure 2 and Figures S2 and S3). The MoFe protein of wild‐type nitrogenase purified from *Azotobacter vinelandii*, an aerobic diazotroph (Figure S4), was electrically wired within the P(GMA‐MMA‐PEGMA)‐NR redox polymer matrix. Bioelectrosynthetic reduction of azide (N_3_^−^), nitrite (NO_2_^−^), and dinitrogen (N_2_) to NH_3_ was achieved by mediated bioelectrocatalysis catalyzed by the MoFe protein via polymer‐bound neutral red in the absence of the Fe protein of the wild‐type nitrogenase.

## Results and Discussion

**Figure 3:**
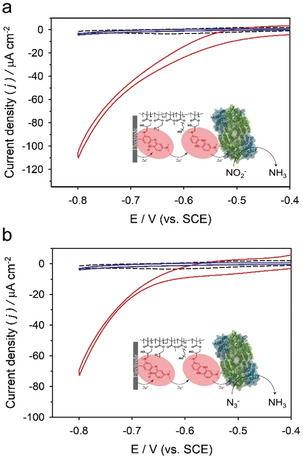
Representative cyclic voltammograms of MoFe/P(GMA‐MMA‐PEGMA)‐NR electrodes (red line) in the presence of a) 100 mm NO_2_^−^ and b) 100 mm N_3_^−^ compared with control bioelectrodes comprising denatured MoFe/P(GMA‐MMA‐PEGMA)‐NR (black dashed line) and active MoFe without P(GMA‐MMA‐PEGMA)‐NR (blue line), respectively. All experiments were performed anaerobically (<1 ppm O_2_) using 100 mm MOPS buffer (pH 6.0) as supporting electrolyte at a scan rate of 5 mV s^−1^.

Cyclic voltammetry (CV) was used to investigate the bioelectrocatalytic properties of the P(GMA‐MMA‐PEGMA)‐NR‐immobilized nitrogenase on the carbon paper electrode as well as control bioelectrodes. The reductive peak potential of pristine P(GMA‐MMA‐PEGMA)‐NR was −0.52±0.01 V vs. SCE (Figure S5), which is more negative than the formal potential for the P cluster (−0.48 V vs. SCE),^11^ suggesting that Δ*E* between the P cluster in the MoFe protein and P(GMA‐MMA‐PEGMA)‐NR is favorable for an mediated electron transfer (MET). The electroenzymatic reaction was evaluated upon injection of 100 mm NaNO_2_ as a water‐soluble nitrogenase substrate into the electrolyte. Cyclic voltammograms of MoFe/P(GMA‐MMA‐PEGMA)‐NR films indicated that P(GMA‐MMA‐PEGMA)‐NR was indeed able to facilitate mediated electrocatalytic reduction of NO_2_^−^ to NH_3_ via the MoFe protein, since a redox event was observed with a significant increase in the reductive current by −110 μA cm^−2^ at −0.8 V (Figure 3 a and Figure S6).

The control bioelectrodes (i.e., denatured MoFe/P(GMA‐MMA‐PEGMA)‐NR and BSA/P(GMA‐MMA‐PEGMA)‐NR) were evaluated under identical conditions (Figure 3 a and Figure S7) to confirm that the enhancement in catalytic current attributed to NO_2_^−^ reduction is only possible with the active MoFe protein/P(GMA‐MMA‐PEGMA)‐NR. Cyclic voltammograms of both control electrodes exhibited insignificant reductive currents compared with electrodes modified with the active MoFe protein/P(GMA‐MMA‐PEGMA)‐NR in the presence of NO_2_^−^. In addition, MoFe/P(GMA‐MMA‐PEGMA)‐NR exhibited electrocatalytic activity to N_3_^−^, another water‐soluble nitrogenase substrate, resulting in a reductive catalytic current due to MoFe‐catalyzed reduction of N_3_^−^ (Figure 3 b and Figure S8). Control bioelectrodes showed no electrocatalytic signal under identical conditions (Figure 3 b and Figure S9). Accordingly, these results suggested that stable and efficient electric wiring of nitrogennase within the redox polymer matrix is achieved, supporting mediated bioelectrocatalysis catalyzed by the MoFe protein via the polymer‐bound neutral red redox relays.

**Figure 4:**
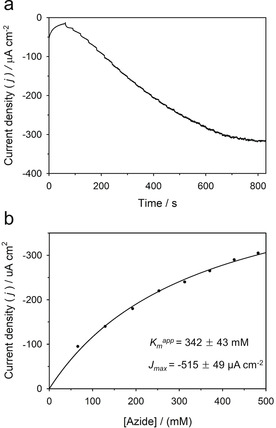
a) Representative chronoamperogram for the reduction of N_3_^−^ by means of MoFe/P(GMA‐MMA‐PEGMA)‐NR modified bioelectrodes in stirred MOPS buffer (pH 6.0, 100 mm) (*E*_applied_=−0.8 V vs. SCE). Substrate injection was started at 60 s and several NaN_3_ additions were performed successively. b) Apparent Michaelis–Menten kinetics of the MoFe/P(GMA‐MMA‐PEGMA)‐NR modified bioelectrode.

Steady‐state amperometric studies were conducted to determine the affinity of the MoFe/P(GMA‐MMA‐PEGMA)‐NR bioelectrode for N_3_^−^ (Figure 4 a). The applied potential was selected based on the voltammetric analysis to supply enough reducing power while minimizing overpotential. Successive injections of N_3_^−^ into the electrolyte resulted in a rapid increase in reduction current, which is due to the electroenzymatic reduction of N_3_^−^ by the MoFe/P(GMA‐MMA‐PEGMA)‐NR active layer. The catalytic current density versus the corresponding N_3_^−^ concentration was analyzed by fitting the Michaelis–Menten kinetic model using nonlinear regression. The apparent Michaelis constants (*K*_M_) for N_3_^−^ and maximum current density (*J*_max_) were calculated to be 342±43 mm N_3_^−^ and −515±49 μA cm^−2^, respectively (Figure 4 b). Substrate diffusion inside the polymer film can cause a change in the linear range of the current response as a function of the concentration.^28^ Amperometric studies using control bioelectrodes (denatured MoFe/P(GMA‐MMA‐PEGMA)‐NR and BSA/P(GMA‐MMA‐PEGMA)‐NR and active MoFe in the absence of the P(GMA‐MMA‐PEGMA)‐NR) under identical steady‐state conditions displayed no bioelectrocatalytic currents upon injections of N_3_^−^ into the electrolyte (Figure S10).

Bulk bioelectrosynthesis was performed using MoFe/P(GMA‐MMA‐PEGMA)‐NR modified electrodes to validate electroenzymatic conversion of N_3_^−^ and NO_2_^−^ to NH_3_. Injections of N_3_^−^ and NO_2_^−^ into the electrolyte solution increased catalytic reduction currents attributed to mediated bioelectrocatalysis by the MoFe/P(GMA‐MMA‐PEGMA)‐NR modified electrode. A fluorimetric assay using *ortho*‐phthalaldehyde and 2‐mercaptoethanol was performed using aliquots of the resulting electrolyte solutions to confirm and quantify NH_3_ formation (Figure S11). The theoretical amount of NH_3_ production from N_3_^−^ and NO_2_^−^ was calculated based on the charge passed during electrosynthesis.

In the case of N_3_^−^ reduction, two different possible N_3_^−^ reduction pathways were used to calculate the maximum faradaic efficiency for NH_3_ productions^26^, ^29^ (Table S1). A value of 94±12 nmol NH_3_ was obtained by N_3_^−^ reduction using the MoFe/P(GMA‐MMA‐PEGMA)‐NR modified electrode for 30 min (Table S1), corresponding to a faradaic efficiency of 95±28 % based on the theoretical maximum amount of 104±18 nmol NH_3_. Under the same experimental conditions, electrosynthetic reduction of NO_2_^−^ resulted in the formation of 17±2 nmol NH_3_, which corresponds to a faradaic efficiency of 67±11 % based on a theoretical amount of NH_3_ of 26±8 nmol NH_3_. The results from bulk electrolysis using a denatured MoFe/P(GMA‐MMA‐PEGMA)‐NR electrode under the same turnover conditions were used as a baseline for the fluorometric assay for NH_3_ determination.

**Figure 5:**
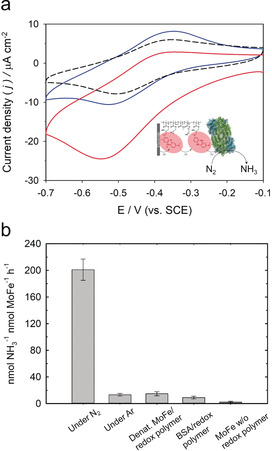
a) Representative cyclic voltammograms of MoFe/P(GMA‐MMA‐PEGMA)‐NR electrodes under Ar (blue), N_2_ with active (red line) or denatured MoFe/P(GMA‐MMA‐PEGMA)‐NR modified electrodes (black dashed line) at scan rates of 5 mV s^−1^. b) The amount of NH_3_ produced after bioelectrosynthesis under ultra‐high‐purity N_2_ or Ar using MoFe/P(GMA‐MMA‐PEGMA)‐NR modified electrodes at −0.7 V vs. SCE for 24 h. Control bioelectrodes (denatured MoFe/P(GMA‐MMA‐PEGMA)‐NR, BSA/P(GMA‐MMA‐PEGMA)‐NR, and MoFe without P(GMA‐MMA‐PEGMA)‐NR) were tested under N_2_ at identical conditions. All bioelectrocatalytic experiments were performed anaerobically (<1 ppm O_2_) using 100 mm MOPS buffer (pH 6.0) as supporting electrolyte.

To further investigate electroenzymatic N_2_ reduction to NH_3_, cyclic voltammetric experiments were performed using the MoFe/P(GMA‐MMA‐PEGMA)‐NR modified electrodes (Figure 5). Cyclic voltammograms measured under an atmosphere of ultra‐high‐purity N_2_ exhibited increasing reductive currents at potentials of −0.38 vs. SCE, while a significant increase in the current density to −25 μA cm^−2^ was obtained at −0.55 V (Figure 5a, red line)as compared with control electrodes equipped with denatured enzyme (Figure 5 a, black dashed line)).^10^, ^11^, ^30^ To determine NH_3_ production from N_2_ fixation, a reductive potential of −0.7 V vs. SCE was chosen based on CV measurements to apply a sufficiently high overpotential. Ultra‐high‐purity N_2_ was injected for 20 min into the electrochemical cell, which was assembled under strictly anaerobic conditions, and the bioelectrosynthesis was conducted for ≈24 h; the reductive current stabilized within ≈1 h. The fluorometric assay using aliquots from the resulting electrolyte solution revealed electrosynthetic N_2_ reduction under formation of 201±16 nmol NH_3_^−1^ nmol MoFe^−1^ h^−1^. Control experiments using the same electrode under Ar or denatured MoFe/P(GMA‐MMA‐PEGMA)‐NR modified electrodes under ultra‐high‐purity N_2_ produced 13±2 nmol NH_3_^−1^ nmol MoFe^−1^ h^−1^ and 15±3 nmol NH_3_^−1^ nmol MoFe^−1^ h^−1^, respectively (Figure 5 b). The results obtained from bulk electrolysis using denatured MoFe electrodes without P(GMA‐MMA‐PEGMA)‐NR or denatured MoFe/P(GMA‐MMA‐PEGMA)‐NR modified electrodes at the same applied potentials were used for baseline correction for the corresponding experiments with the active enzyme.

**Figure 6:**
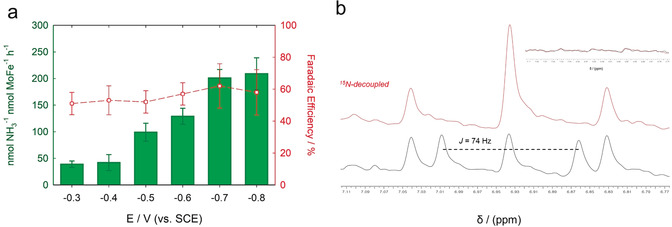
a) Amount of produced NH_3_ (green) and faradaic efficiency (red) in dependence on the applied potentials under ultra‐high‐purity N_2_‐enriched conditions using MoFe/P(GMA‐MMA‐PEGMA)‐NR. b) ^1^H NMR spectroscopic determination of ^15^NH_3_ produced by bioelectrosynthesis of ^15^N_2_ using MoFe/P(GMA‐MMA‐PEGMA)‐NR modified electrodes at −0.8 V (vs. SCE) under ^15^N‐non‐decoupled (black) and ^15^N‐decoupled (red) conditions. The inset shows that the characteristic signal pattern for ^15^NH_3_ was absent in electrolytes from experiments using denatured MoFe/P(GMA‐MMA‐PEGMA)‐NR. All bioelectrocatalytic experiments were performed anaerobically (<1 ppm O_2_) using 100 mm MOPS buffer (pH 6.0) as the electrolyte.

The NH_3_ production yield and faradaic efficiency in dependence on the applied potential were explored under ultra‐high‐purity N_2_ enriched conditions (Figure 6 a). Aliquots of the resulting electrolyte solutions were analyzed by fluorescence spectroscopy, confirming that N_2_ reduction to NH_3_ was attained at potentials as high as −0.3 V, and the maximum yield of NH_3_ was achieved at an applied potential of −0.8 V. This result suggests that at a sufficiently high applied overpotential the majority of the polymer‐tethered NR moieties would be in their reduced state following the Nernst equation, leading to a high electrocatalytic driving force consistent with Butler–Volmer kinetics.^20^, ^26^, ^31^, ^32^

Experiments with ^15^N isotope labeled N_2_ were performed to provide further evidence for bioelectrosynthetic N_2_ turnover. Bulk electrolysis using a MoFe/P(GMA‐MMA‐PEGMA)‐NR modified electrode using isotopically enriched ^15^N_2_ was conducted and the presence of ^15^NH_3_ in the resulting electrolysis solution was confirmed by NMR (nuclear magnetic resonance) spectroscopy (Figure 6 b). The ^1^H NMR spectra showed a signal with a coupling constant of 74 Hz, attributed to ^15^NH_3_, which converged to a singlet at 6.95 ppm in the ^15^N decoupling experiment.^11^, ^33^ The characteristic signal pattern for ^15^NH_3_ was absent when control bioelectrodes were used (i.e., denatured MoFe/P(GMA‐MMA‐PEGMA)‐NR and MoFe electrodes without P(GMA‐MMA‐PEGMA)‐NR). NMR spectra of the electrolyte solution before the electrochemical experiments revealed a background ^14^NH_3_ signal, reiterating the significance of the ^15^N isotope labeled NMR experiments for identifying bioelectrosynthesized NH_3_. ^1^H NMR analysis unequivocally demonstrated that biosynthetic N_2_ reduction to NH_3_ is enabled by the P(GMA‐MMA‐PEGMA)‐NR‐immobilized MoFe protein system.

## Conclusion

A metal‐free redox‐polymer‐based electroenzymatic nitrogen fixation system providing stable and efficient electric wiring of nitrogenase within a low‐potential neutral‐red‐modified polymer matrix enabled mediated bioelectrocatalysis of N_3_^−^, NO_2_^−^, and N_2_ under formation of NH_3_ catalyzed by the MoFe protein via the polymer‐bound redox moieties distributed in the polymer matrix in the absence of the Fe protein. Bioelectrosynthetic N_2_ fixation to NH_3_ was confirmed by ^15^N_2_ labeling experiments and NMR analysis. As the proposed all organic P(GMA‐MMA‐PEGMA)‐NR polymer have not yet been studied in combination with nitrogenases for electrosynthetic NH_3_ synthesis, the present research provides a first step for future bioelectrosynthetic applications.

## Conflict of interest

The authors declare no conflict of interest.

## Supporting information

As a service to our authors and readers, this journal provides supporting information supplied by the authors. Such materials are peer reviewed and may be re‐organized for online delivery, but are not copy‐edited or typeset. Technical support issues arising from supporting information (other than missing files) should be addressed to the authors.

Supplementary
