# Supplementary material for: Electroenzymatic Nitrogen Fixation Using a MoFe Protein System Immobilized in an Organic Redox Polymer
Source: Angew Chem Int Ed Engl. 2020 Jul 22;59(38):16511–6. doi: 10.1002/anie.202007198 (PMC7540466; doi:10.1002/anie.202007198)
Supplement: Supplementary file 1 — Supplementary [file ANIE-59-16511-s001.pdf]

Angewandte Chemie
Eine Zeitschrift der Gesellschaft Deutscher Chemiker

Supporting Information

Electroenzymatic Nitrogen Fixation Using a MoFe Protein System Immobilized in an Organic Redox Polymer

Yoo Seok Lee, Adrian Ruff, Rong Cai, Koun Lim, Wolfgang Schuhmann, and Shelley D. Minteer**

anie_202007198_sm_miscellaneous_information.pdf

Supporting Information

Table of Contents

Experimental Procedures 2
Supporting Figures (S1-S11) 4
Supporting Tables (S1) 7
References 7
Author Contributions 7

Experimental Procedures

Materials

All chemicals and materials were purchased from Alfa-Aesar, Sigma Aldrich, VWR, and Acros Organics and were used as received except otherwise noted. The monomers glycidyl methacrylate (GMA), methyl methacrylate (MMA) and poly(ethylene glycol methacrylate) (PEGMA, Mn = 500, dissolved in *iso*-propyl alcohol, 0.052 g mL^{-1}) were passed to a short column filled with an inhibitor remover and stored at -20 °C (GMA, MMA) or room temperature (PEGMA). The initiator AIBN (azobisisobutyronitrile) was recrystallized from hot toluene or hot methanol.[1] CD_3CN for NMR spectroscopy was purchased from Deutero and stored at 4° C. For the modification of the polymer backbone P(GMA-MMA-PEGMA) with neutral red, the free base was used which was precipitated from an aqueous solution by adding a base. The free base (red-orange powder) was dried in an oven and stored at room temperature.

Methods

All NMR experiments were conducted with a DPX250 spectrometer from Bruker with a ^1H resonance frequency of 250.13 MHz. All measurements were conducted at room temperature. Samples were filtered to remove particles. All UV-vis measurements were conducted with a Cray60 spectrophotometer from Agilent in quartz cuvettes with an optical path length of 1 cm. Electrochemical characterization of the redox polymer was performed with a PalmSens2 v4.4 (PalmSens) potentiostat with a three-electrode configuration in standard electrochemical glass cells. Polymer modified electrodes were prepared by a standard drop cast process and dried for several our prior to measurement. Size exclusion chromatography (SEC) was performed against polystyrene (PS) standards in THF at 30 °C. Data from the RI detector were analyzed with the PSS WinGPC Unity software. Sample concentration was 15 mg mL^{-1}.

Synthesis of the polymer backbone poly(glycidyl methacrylate-*co*-methyl methacrylate-*co*-poly(ethylene glycol) methacrylate)), P(GMA-MMA-PEGMA)

The synthesis of the polymer backbone P(GMA-MMA-PEGMA) was conducted following procedures as described in ref.[2] The monomers GMA (500.16 mg, 3.52 mmol), MMA (280.8 mg, 2.81 mmol) and PEGMA (0.052 g mL^{-1}, 6.73 mL, 350 mg, 0.7 mmol) were dissolved in THF (5 mL) under argon atmosphere. The radical initiator AIBN (4.9 mg, 0.03 mmol) was added and the reaction mixture was heated to 70 °C under an argon atmosphere (closed Schlenk tube, connected to a pressure release valve). The transparent and colorless reaction mixture was stirred for 285 min. The solution was cooled down to room temperature and the product was precipitated by adding 120 mL of *n*-pentane to yield the crude polymer as a colorless solid. The turbid solution was decanted off and centrifuged and the precipitate was combined with the colorless crude polymer. The product was re-dissolved in THF (≈15 mL) and precipitated again by adding n-pentane. The solution was again decanted off and centrifuged and the purification step was again repeated. The combined solids were air dried and the remaining solvent was removed by shortly applying a vacuum. The solid was immediately dissolved in MeCN (20 mL) and stored as a stock solution with a concentration of 19 mg mL^{-1} at room temperature. Yield: 380 mg (33 %). ^1H-NMR (250.13 MHz, CD_3CN) δ/ppm: 4.32 and 4.27 (duplet, GMA -CH_2-O), 4.05 (s, PEGMA -CH_2-O), 3.69 (broad, overlapping, GMA -CH_2-O), 3.54 (singlet, overlapping, MMA -OMe, PEGMA -CH_2-CH_2-), 3.18, 2.78 and 2.62 (all singlet, epoxide in GMA), 1.00 (s, -CH_3 backbone), 0.83 (singlet, -CH_3 backbone). Composition via integral ratio of the CH_2-O group in GMA (1 H) at 4.3 ppm (1), the CH_2-O-CO- group in PEGMA (2 H) at around 4.05 ppm (0.32) and the CH_3 groups at 1.0 and 0.8 ppm (5.47 H, 1.99 for MMA; GMA = 3 H and PEGMA = 0.48) was estimated to be GMA:MMA:PEGMA = 55 mol% : 36 mol% : 9 mol% (nominal composition = GMA:MMA:PEGMA = 50 mol% : 40 mol% : 10 mol%). SEC against PS standard, THF): Mn = 4 kDa, PDI 2.3.

Synthesis of the redox-active polymer neutral red modified poly(glycidyl methacrylate-*co*-methyl methacrylate-*co*-poly (ethylene glycol) methacrylate), P(GMA-MMA-PEGMA)-NR

Under an argon atmosphere, neutral red (free base, 36.25 mg, 0.13 mmol, 0.46 eq with respect to the GMA amount in the backbone) was suspended in 5 mL of DMSO, and the organic base NEt_3 (21.9 mg, 0.22 mmol) was added. The slurry was stirred until the base was fully dissolved. Then, P(GMA-MMA-PEGMA) solution (19 mg mL^{-1} in MeCN, 5.25 mL, 99.75 mg, 0.28 mmol GMA units assuming 49 wt% of GMA within the backbone) was slowly added with a syringe. The resulting mixture was heated to 60 °C and stirred for two days in a closed vessel equipped with a pressure release valve. The brown reaction mixture was cooled to room temperature, and most of the organic solvent was removed under vacuum. The remaining solution was quenched with water and acidified with a few drops of diluted aqueous HCl (protonation of the neutral red moieties to increase water solubility, color changes from brown to red). The crude mixture was purified by membrane filtration using membrane filters with a molecular weight cutoff of 10 kDa (VivaSpin, Turbo 15, Sartorius) and centrifugation. The aqueous workup was stopped when the washing solution became colorless. The polymer was obtained as a red-brown aqueous solution with a concentration of 17 mg mL^{-1}. Note that the secondary amine that is formed after binding of NR to the backbone shows a higher nucleophilicity than the primary amine in the free base and might thus react with a second epoxide function (intra- and/or interpolymeric reaction). Hence, the exact degree of binding of the NR moiety is not known (indicated by the dashed line). Redox potential E vs. Ag/AgCl/3 M KCl = -550 mV (drop cast film, 0.1 M phosphate buffer, pH 7.3). UV-vis (DMSO) λ/nm: under neutral conditions (wet DMSO): 285 (max), 461, 548 (free modifier: 276 (max), 543); acidic conditions (DMSO/HCl_{aq}): 286 (max), 450, 534 (free modifier: 276 (max), 543) , basic conditions (DMSO/$NaOH_{aq}$): 282 (max), 452 (free modifier: 280 (max), 457). It should be noted that for clarity reasons only the product of the mono-addition of the NR modifier to the epoxide modified polymer is shown in Figure 2, indicated by the dashed line. The secondary amine formed at the polymer may react with an additional epoxide

function within the backbone. The resulting P(GMA-MMA-PEGMA)-NR shows high electrochemical and physical stability. After 50 continuous CV scans of P(GMA-MMA-PEGMA)-NR at 100 mV s^{-1} the reductive peak currents decreased only by <7%.

Growth of *Azotobacter vinelandii* and purification of nitrogenase

Mo-dependent nitrogenase from *A. vinelandii* was expressed as described previously.[3,4] Briefly, the wild-type cells were cultivated in 18 L modified Burk medium supplemented with 10 mM ammonium (NH_4^+). Then, the cells were collected via centrifugation and resuspended into NH_4^+ free growth media to enhance nitrogenase expression. The cells were regrown until the optical density at 600 nm reached 1.5. All of the following steps were carried out under strictly anaerobic conditions. The cells were lysed by sonication in the presence of 2 mM sodium dithionite (DT). The crude extract was separated by anion exchange chromatography (Q-Sepharose, GE Healthcare) over a linear gradient of sodium chloride (NaCl) from 200 mM to 640 mM to separate the MoFe and FeP proteins. His-tagged MoFe was purified using HisTrap HP column (GE Healthcare) and were desalted using a Hitrap desalting column equilibrated with 100 mM MOPS and 2 mM DT at pH = 7.0. The purified protein was concentrated to ≥ 20 mg mL^{-1} and was shock-frozen in liquid N_2 until further use. The purity of the MoFe proteins was greater than 95% based on the SDS-PAGE analysis using Coomassie blue staining. The purity of the protein was verified by SDS-PAGE gel (Figure S3). Pure protein was concentrated to ~20 mg/mL.

MoFe protein/P(GMA-MMA-PEGMA)-NR bioelectrode preparation

Growth of *Azotobacter vinelandii* and purification of nitrogenase are shown in Figure S3. The purified MoFe protein solution (5 μL, 25 mg mL^{-1} in a 100 mM MOPS buffer, pH 7.0) was mixed via vortex for 10 s with 10 μL of P(GMA-MMA-PEGMA)-NR solution (10 mg mL^{-1} in H_2O). To the polymer/protein mixture, a solution of crosslinker (AZO) (1 μL, 2.7% by volume in H_2O) was vortexed for 10 s. Non-wet-proofed carbon paper (AvCarb MGL 190) was cut into 3 cm x 0.5 cm strips. AvCarb MGL 190: thickness (0.19 mm), bulk density (0.44 g/cm^3), porosity (78%), electrical resistivity (75 mΩcm) and no PTFE treatment. These strips were dipped into melted paraffin wax to obtain a 0.5 cm x 0.5 cm square electrode that remained exposed at one end. The MoFe/P(GMA-MMA-PEGMA)-NR/AZO mixture (10 μL) was then drop-coated onto the carbon paper electrode (AvCarb MGL 190) and then dried under an atmosphere of Ar/H_2 (3.2%) at room temperature for 10 h. Control bioelectrodes were made with an equivalent loading of denatured MoFe protein (by mass) or BSA (bovine serum albumin) replacing the catalytically active MoFe protein. The blank was made with equivalent loading of P(GMA-MMA-PEGMA)-NR (by mass) without MoFe protein.

Electrochemical analysis

All electrochemical analyses were performed anaerobically (<1 ppm O_2) to avoid competitive O_2 reduction at the working electrode and to preserve the activity of the MoFe nitrogenase. pH 6.0 100mM MOPS buffer was degassed and used as the supporting electrolyte to avoid migration issues associated with N_3^- and NO_2^-. Electrochemical measurements were performed using a CH Instruments potentiostat model 1230b, with a saturated calomel reference electrode (SCE), a platinum mesh counter electrode, and the carbon paper working electrode (0.5 cm x 0.5 cm) in a standard 3-electrode configuration. Electrolysis was carried out at a constant potential of either −0.3, −0.4, −0.5, −0.6, −0.7 or −0.8 V vs SCE with rapid stirring (400 rpm) using 100 mM MOPS buffer, pH 6.0 at 25 ºC. All voltammograms are plotted in IUPAC notation with cathodic currents being negative.

Ammonia/ammonium fluorescence detection assay

The produced NH_3 was quantified as previously reported.[3, 5, 6] A reagent to detect NH_3 was prepared by mixing 100 mL phosphate buffer (pH 7.3, 200 mM), 25 μL of 2-mercaptoethanol, and *ortho*-phthalaldehyde (270 mg dissolved in 5 mL of ethanol, Figure S4). Note: this reagent is light-sensitive and it has to be used and stored in the absence of light. Following bioelectrosynthesis experiments, 250 μL of the electrolyte solution containing the formed NH_3 was mixed with 1000 μL of the premade reagent for NH_3 detection. The mixture was incubated for 30 min. The produced NH_3 was quantified by measuring the fluorescence emission at 472 nm when excited at a wavelength of 410 nm. All samples were triplicated and corrected against background samples prepared with BSA or denatured MoFe protein bioelectrodes.

NMR analysis

^{15}N labeled experiments were conducted to verify that NH_3 is produced electrochemically. Bulk electrolysis cells were assembled under an Ar/H_2 (3.2%) atmosphere. Experiments performed under $^{15}N_2$ were carried out in a sealed three-neck flask. Electrolysis cells were degassed after assembly under vacuum at room temperature for 1 h to ensure all H_2 was removed and then filled with $^{15}N_2$. All electrolysis experiments were carried out for 24 h. Electrolysis experiments were performed under $^{15}N_2$ from Sigma Aldrich (MDL # MFCD00084232, >98% isotopic purity purity ^{15}N; 99% (CP) assay). Gases were purged through sequential solutions of 0.1 M HCl and 10 mM sodium dithionite to trap any contaminant NH_3 and O_2, respectively. After electrolysis experiments, samples were first spun through a 10 kDa MWCO membrane to remove any leached protein. The NMR spectra were collected at room temperature on a 500 MHz (^1H) Varian Inova spectrometer equipped with a cryoprobe. ^1H 1-D spectra were collected using a selective excitation pulse for water suppression and a spectral width of 8012.8 Hz (16.0 ppm). Spectra were collected both with and without ^{15}N decoupling during acquisition to demonstrate the absence or presence of ^{15}N-enriched molecules. ^1H-NMR signals were averaged over 24 h in all cases, and baseline subtraction was utilized to account for the overlapping residual peak corresponding to water (after solvent suppression).

Results and Discussion

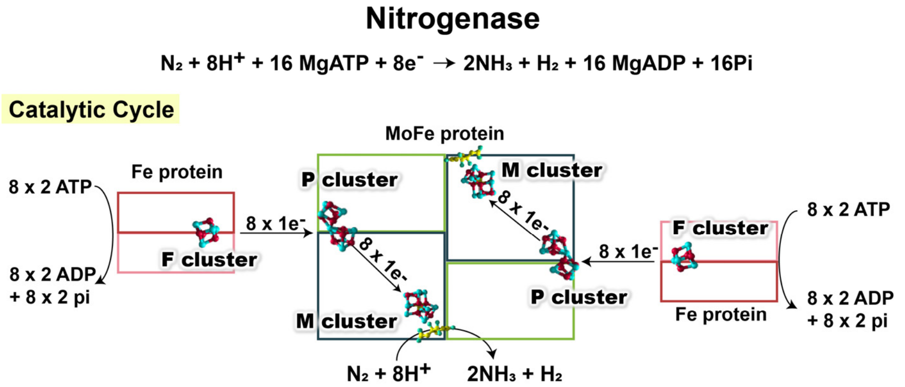

Figure S1. The nitrogenase catalytic cycle illustrating N_2 reduction involving the transient binding of the Fe protein with the MoFe protein and ATP hydrolysis..

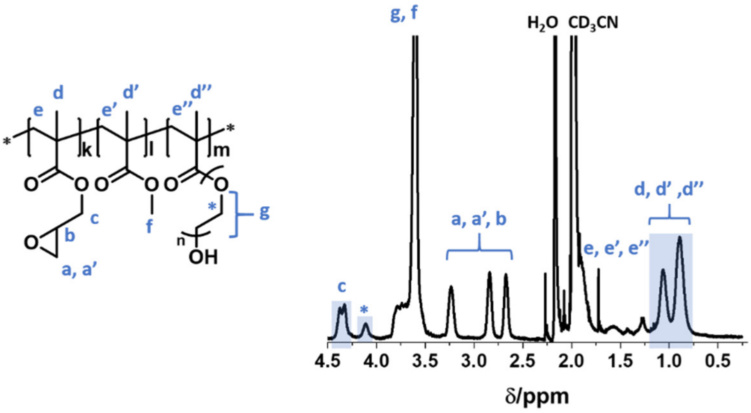

Figure S2: ^{1}H-NMR (250.13 MHz, CD_3CN) of the polymer backbone P(GMA-MMA-PEGMA), including the signal assignment that is attached to the molecular structure. Blue shaded areas indicate signals/integrals that were used for the estimation of the actual composition of the polymer (GMA:MMA:PEGMA = 55:36:9 mol%; nominal composition: 50:40:10 mol%). * indicates the methylene moiety in PEGMA that is directly attached to the ester function of the monomer.

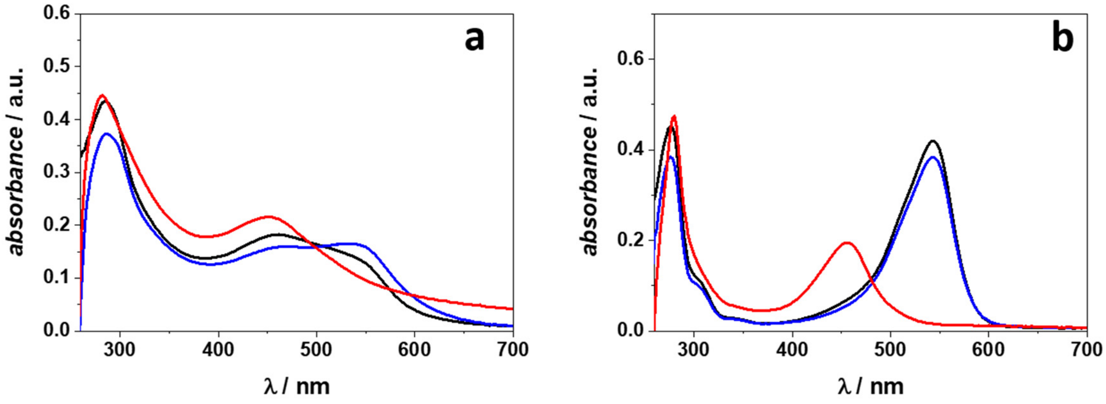

Figure S3: UV-Vis of the redox polymer P(GMA-MMA-PEGMA)-NR (a) and the free Neutral red (b) under neutral (black traces), acidic (blue traces) and basic (red traces) DMSO. The absorption maxima at around 280 nm of the polymer-bound NR species show the same behavior as the free modifier. However, the signature of the signals between 400 to 600 nm differ. This might be an effect of the transition of the primary amino group in the free NR to a secondary/tertiary amino group in the polymer-bound system.

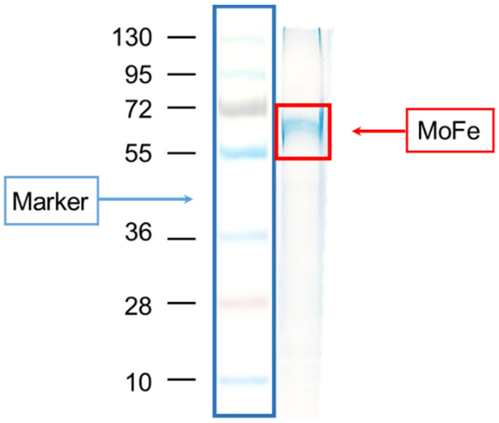

Figure S4. SDS-PAGE analysis of purified MoFe protein.

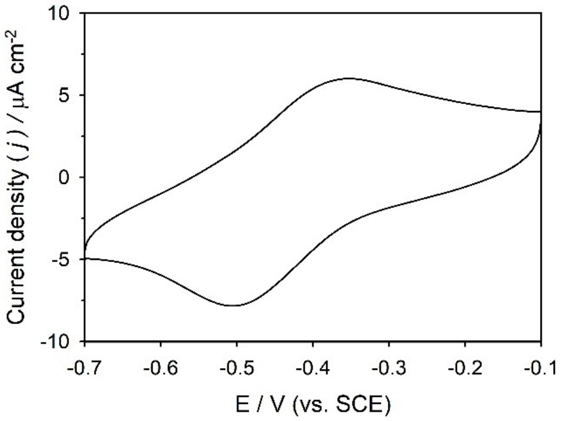

Figure S5. Cyclic voltammetry of pristine P(GMA-MMA-PEGMA)-NR electrode (P(GMA-MMA-PEGMA)-NR coated electrode without enzyme) evaluated at a scan rate of 10 $mV{\cdot}s^{-1}$ in 100 mM MOPS buffer (pH 6). The redox polymer shows a chemically reversible signal with a mid-point potential of around -450 mV *vs.* SCE.

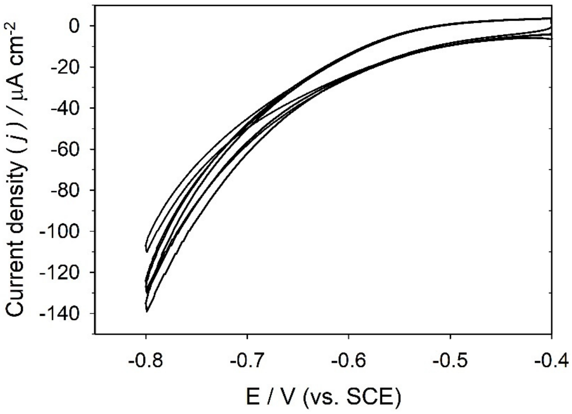

Figure S6. Cyclic voltammograms of a MoFe/P(GMA-MMA-PEGMA)-NR electrode at a scan rate of 5 $mV{\cdot}s^{-1}$ (2 consecutive CVs of 2 cycles in 100 mM MOPS buffer (pH 6) in the presence of 100 mM NO_2^-). The voltammetric experiment was performed under anaerobic conditions with the O_2 concentration <1 ppm at room temperature.

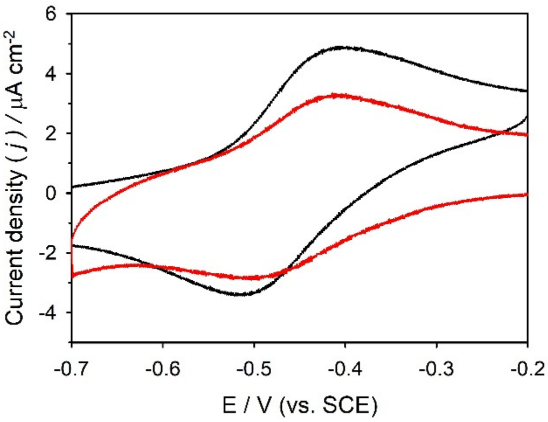

Figure S7. Cyclic voltammetry of BSA/P(GMA-MMA-PEGMA)-NR (red line) and electrodes modified with pristine redox polymer (black) evaluated at a scan rate of 5 mV·s^{-1} in 100 mM MOPS buffer (pH 6) in the presence of 100 mM NO_2^-. The voltammetric experiments were performed under anaerobic conditions where the O_2 concentration was continuously <1 ppm at room temperature.

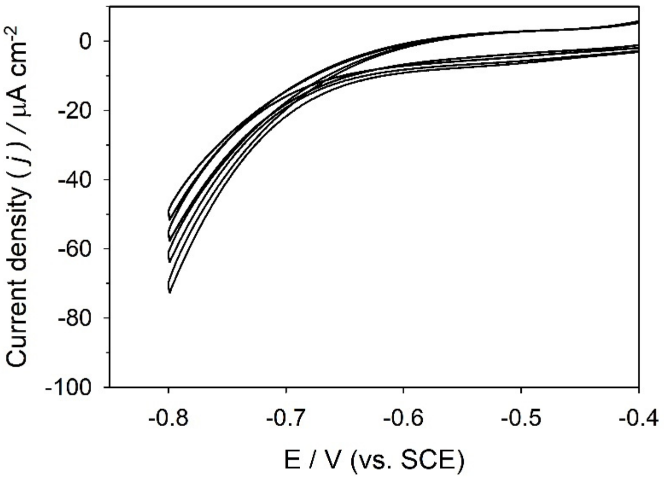

Figure S8. *Cyclic voltammograms of a MoFe/P(GMA-MMA-PEGMA)-NR electrode at a scan rate of 5 mV·s^{-1} (2 consecutive CVs of 2 cycles in 100 mM MOPS buffer (pH 6) in presence of 100 mM N_3^-). The voltammograms were recorded under anaerobic conditions with an O_2 concentration <1 ppm at room temperature.*

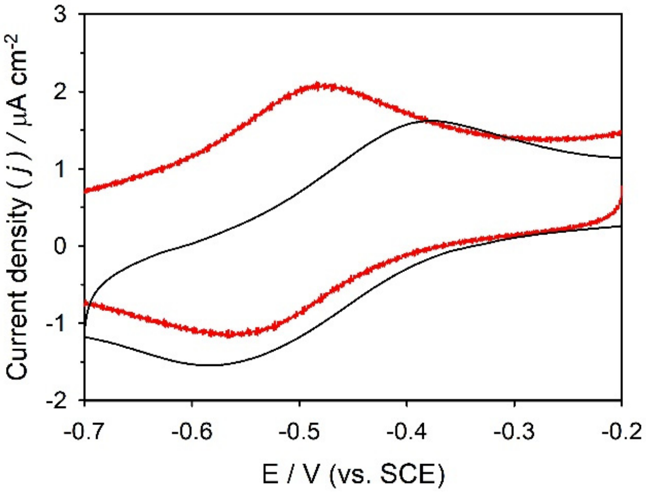

Figure S9. Cyclic voltammetry of BSA/P(GMA-MMA-PEGMA)-NR (red line) and electrodes modified with pristine redox polymer (black) evaluated at a scan rate of 5 mV·s^{-1} in 100 mM MOPS buffer (pH 6) in the presence of 100 mM N_3^-. The voltammetric experiments were performed under anaerobic conditions where the O_2 concentration was continuously <1 ppm at room temperature.

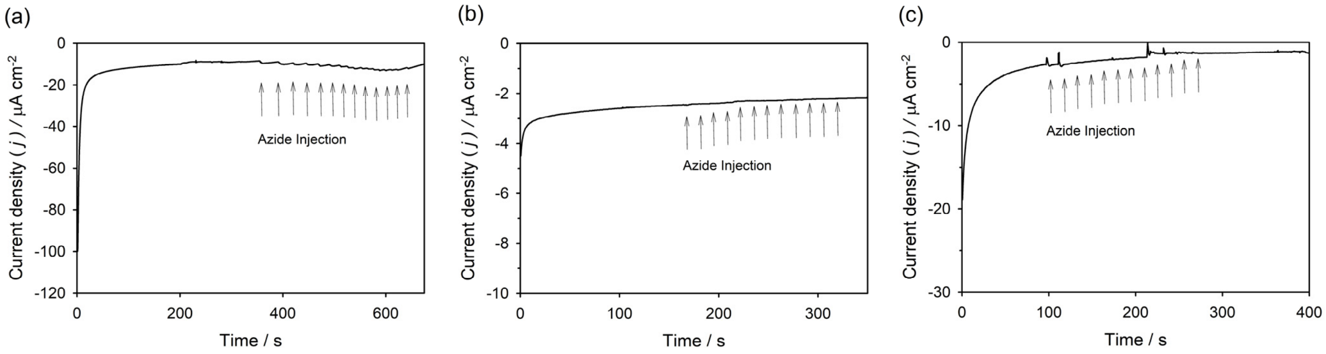

Figure S10. Bulk bioelectrosynthetic reduction of N_3^- by a control bioelectrode in stirred MOPS (pH 6.0, 100 mM) ($E_{applied}$ = -0.8 V *vs.* SCE). Successive injections of N_3^- were made from a stock solution. Control bioelectrodes consist of (a) denatured MoFe/ P(GMA-MMA-PEGMA)-NR, (b) BSA/ P(GMA-MMA-PEGMA)-NR, and (c) active MoFe without P(GMA-MMA-PEGMA)-NR. Each arrow indicates a start of slow azide injection for 15 sec. MoFe protein was denatured by exposure to air for 1 day. Reactions depicted in (a), (b) and (c) are unbalanced.

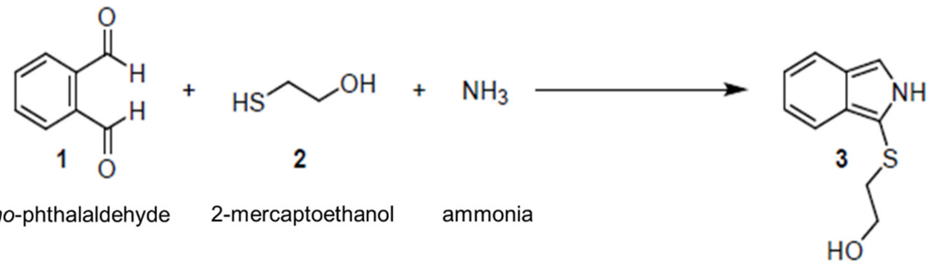

Figure S11. Fluorescence assay for NH_3 quantification; NH_3 detection reagent consists of 2-mercaptoethanol and *ortho*-phthalaldehyde forms a fluorescent complex with NH_3.[3]

Table S1. Calculated Faradaic efficiencies for N_3^- and NO_2^- reduction by MoFe/P(GMA-MMA-PEGMA)-NR bioelectrodes. The theoretical maximum quantity of NH_3 anticipated to be generated as a function of the electron transferred during the electrosynthesis was also calculated based on the reaction equations. Bioelectrosynthesis was performed for 30 min at −0.8 V *vs.* SCE in MOPS buffer (100 mM, pH 6).[7, 8]

Reduction reaction	NH_3 detected (nmol)	NH_3 calculated (nmol)	Faradaic efficiency (%)
$N_3^- + 3H^+ + 2e^- \rightarrow N_2 + NH_3$	94 ± 12	138 ± 24	69 ± 24
$N_3^- + 9H^+ + 8e^- \rightarrow 3NH_3$		104 ± 18	95 ± 28
$NO_2^- + 7H^+ + 6e^- \rightarrow NH_3 + 6H_2O$	17 ± 2	26 ± 8	67 ± 11

References

[1] J. Szczesny, N. Marković, F. Conzuelo, S. Zacarias, I. A. Pereira, W. Lubitz, N. Plumeré, W. Schuhmann, A. Ruff, *Nature Comm.* **2018**, *9*, 1-9
[2] F. Lopez, S. Zerria, A. Ruff, W. Schuhmann, *Electroanal.* **2018**, *30*, 1311-1318.
[3] D. P. Hickey, R. Cai, Z.-Y. Yang, K. Grunau, O. Einsle, L. C. Seefeldt, S. D. Minteer, *J. Amer. Chem. Soc.* **2019**, *141*, 17150-17157.
[4] R. D. Milton, R. Cai, S. Sahin, S. Abdellaoui, B. Alkotaini, D. Leech, S. D. Minteer, *J. Amer. Chem. Soc.* **2017**, *139*, 9044-9052.
[5] M. Duca, J. R. Weeks, J. G. Fedor, J. H. Weiner, K. A. Vincent, *ChemElectroChem* **2015**, *2*, 1086-1089.
[6] R. M. Holmes, A. Aminot, R. Kérouel, B. A. Hooker, B. J. Peterson, *Can. J. Fish. Aquat. Sci.* **1999**, *56*, 1801-1808..
[7] R. D. Milton, S. Abdellaoui, N. Khadka, D. R. Dean, D. Leech, L. C. Seefeldt, S. D. Minteer, *Energ. Environ. Sci.* **2016**, *9*, 2550-2554.
[8] J. F. Rubinson, B. K. Burgess, J. L. Corbin, M. J. Dilworth, *Biochem.* **1985**, *24*, 273-283.

Author Contributions

YSL, AR, WS and SDM conceived the study and designed the experiments. AR and WS designed and synthesized the redox polymer. YSL produced the Mo-nitrogenase and performed all further experiments. YSL and SDM wrote the manuscript. All authors edited the manuscript.
